# Antiresorptive effect of a cathepsin K inhibitor ONO-5334 and its relationship to BMD increase in a phase II trial for postmenopausal osteoporosis

**DOI:** 10.1186/s12891-017-1625-y

**Published:** 2017-06-19

**Authors:** Makoto Tanaka, Yoshitaka Hashimoto, Chihiro Hasegawa, Steve Deacon, Richard Eastell

**Affiliations:** 1 Research Promotion, Ono Pharmaceutical Co., Ltd, 3-1-1 Sakurai, Shimamoto, Osaka, 618-8585 Japan; 2 Translational Medicine Center, Ono Pharmaceutical Co., Ltd, Shimamoto, Osaka Japan; 3 Clinical Development, Ono Pharma UK Ltd, London, UK; 4 Academic Unit of Bone Metabolism, University of Sheffield, Sheffield, UK

**Keywords:** Cathepsin K, ONO-5334, Bone resorption, PK/PD relationship, OCEAN study

## Abstract

**Background:**

ONO-5334 is a cathepsin K inhibitor that induced bone mineral density (BMD) gain in a phase II study in postmenopausal osteoporosis patients. Even though the antiresorptive effect could only be monitored in the morning during the study, simulation can allow the antiresorptive effect to be assessed over 24 h, with assessment of the relationship to BMD gain.

**Methods:**

Inhibition of the serum C-telopeptide of type I collagen (sCTX) level at doses of ONO-5334 of 100 mg once daily (QD), 300 mg QD, and 50 mg twice daily (BID) was simulated using plasma ONO-5334 pharmacokinetic (PK) data for repeated dose administration in a phase I study and corresponding sCTX inhibition from the PK-pharmacodynamic (PK/PD) relationship. sCTX was selected because it has a high signal-to-noise ratio compared to other telopeptides. A negative sigmoidal shape for the PK/PD relationship between plasma ONO-5334 and sCTX levels was obtained in our previous study.

**Results:**

The simulated sCTX inhibition reached >99% of the maximal inhibitory effect (*Emax*) at 0.5 h in all treatment groups, and decreased to <80% *Emax* at 8 and 12 h at 50 mg BID and 100 mg QD, respectively. However, sCTX inhibition at 300 mg QD was maintained at ≥82% *Emax* over 24 h. The mean sCTX inhibition rates for 24 h at 100 mg QD, 300 mg QD and 50 mg BID were 63, 95 and 80% *Emax*, respectively. There was a positive linear relationship by treatment group between mean sCTX inhibition over 24 h and observed BMD gain in the phase II study.

**Conclusion:**

The dose response for BMD with ONO-5334 at 100 and 300 mg QD and higher BMD gain at 50 mg BID vs. 100 mg QD can be explained by sCTX inhibition over 24 h. The simulation gave the antiresorptive effect of ONO-5334 over 24 h and allowed prediction of BMD gain due to ONO-5334.

**Trial registration:**

The registration number in The European Union Clinical Trials Register is 2007–002417-39. The date of registration was August 31, 2007.

**Electronic supplementary material:**

The online version of this article (doi:10.1186/s12891-017-1625-y) contains supplementary material, which is available to authorized users.

## Background

Osteoporosis is defined as a systemic skeletal disease characterized by low bone mass and microarchitectural deterioration of bone tissue, with a consequent increase in bone fragility and susceptibility to fracture [1]. The disease is characterized by an imbalance in skeletal turnover such that bone resorption exceeds bone formation, thereby leading to a reduction in overall bone mass [2]. Osteoporosis is usually diagnosed based on bone mineral density (BMD), and several biochemical markers are used to monitor bone resorption and bone formation. Drugs for osteoporosis are classified as antiresorptive drugs that inhibit bone resorption, such as bisphosphonates, and anabolic agents that stimulate bone formation, such as parathyroid hormone [3]. Bisphosphonates are widely used first-line osteoporosis agents due to fracture prevention by BMD increase and prolonged antiresorptive effect after withdrawal [4].

Cathepsin K, a member of the papain cysteine protease superfamily, is released by osteoclasts and degrades collagen of bone [5]. Genetic evidence indicates a critical role of cathepsin K in bone resorption in humans and mice [6–8]. Cathepsin K releases type I collagen crosslinks such as *N-*telopeptides of type I collagen (NTX) and *C-*telopeptides of type I collagen (CTX), which are widely used as diagnostic biomarkers for evaluation of osteoporosis [9–11]. Therefore, several cathepsin K inhibitors have been examined as potential therapy for osteoporosis [12–14]. ONO-5334 is an orally available cathepsin K inhibitor with established efficacy for increasing BMD and proven safety in a 2-year phase II study in osteoporosis (the OCEAN study) [15–17]. In earlier phase I studies, ONO-5334 decreased serum CTX (sCTX) in a dose-dependent manner. Plasma ONO-5334 reached a maximum (Cmax) within 1.5 to 2 h of dosing and thereafter decreased in a biphasic manner, with a rapid initial phase followed by a slower phase. In repeated administration, the plasma ONO-5334 level just before the next dose (trough) reached steady state in 2 days after the first administration. Chemically, ONO-5334 is an α-amino acid derivative with a ketone, which differs from another cathepsin K inhibitor, odanacatib, an aliphatic nitrile [18, 19]. These cathepsin K inhibitors have similar efficacy in clinical and non-clinical studies [15, 16, 20–24], but the pharmacokinetic (PK) profiles were differ, with odanacatib developed as a once weekly treatment [18, 25].

The OCEAN study was a randomized, double-blind, placebo- and active-controlled parallel-group trial performed to investigate the efficacy and safety of 100 mg once daily (QD), 300 mg QD and 50 mg twice daily (BID) ONO-5334 immediate release (IR) tablets [16, 17]. ONO-5334 300 mg QD was chosen because phase I data suggested that ≥300 mg ONO-5334 showed a maximum suppressive effect on sCTX, and that 300 mg ONO-5334 maintained the suppressive effect on sCTX for 24 h after administration. ONO-5334 100 mg QD was added for evaluation of the dose response. ONO-5334 50 mg BID was chosen to compare the efficacy and safety of two regimens with a 100 mg total daily dose, to assess the potential for development of a slow release (SR) formulation. ONO-5334 increased BMD at all measured sites at 50 mg BID and 300 mg QD in the trial. A dose of 100 mg QD had less efficacy for increasing BMD compared to 50 mg BID and 300 mg QD. These findings suggest that a higher trough concentration of ONO-5334, rather than a higher Cmax, is more important for BMD increase. The drug was administered in the evening in the QD groups, and bone turnover markers were assessed only in the morning, based on bone resorption markers showing rhythmic variations and peaking at night [26, 27]. Therefore, evaluation of the antiresorptive effect over 24 h and its relationship to BMD was limited in the OCEAN study. It was also difficult to calculate the antiresorptive effect over 24 h in phase I studies of ONO-5334 IR formulations because of limited sampling points for detection of significant changes in bone resorption markers.

In contrast, ONO-5334 SR formulations most likely maximize the potency of ONO-5334 by reducing Cmax and increasing exposure around the trough [28]. In addition, the flat PK profile of ONO-5334 SR may minimize the lag-time between PK and pharmacodynamic (PD) inhibition of bone resorption markers. Therefore, ONO-5334 SR provided a better evaluation of the PK/PD relationship in our previous study [29]. In an analysis excluding circadian variation of bone resorption markers, the plasma levels of bone resorption markers and ONO-5334 were fitted with sigmoidal maximal inhibitory effect (*Emax*) models, simply reflecting inhibition of cathepsin K. Furthermore, Eastell et al. clearly showed that changes in sCTX inhibition with ONO-5334 SR morning vs. evening dosing parallel changes in the PK profile, highlighting a clear link between PK levels and antiresorptive effects [30].

Even though the antiresorptive effect could only be monitored at one point in the morning in the OCEAN study, simulation can allow the antiresorptive effect to be assessed for 24 h and may provide a better assessment of the relationship of this effect with BMD increase. sCTX has the highest signal-to-noise ratio among serum and urinary NTX and CTX bone resorption markers [29]. In this post-hoc analysis, the duration of antiresorptive effects, sCTX inhibition, and the relationship between antiresorptive effects and increases in BMD were investigated in postmenopausal patients with osteoporosis.

## Methods

### Studies from which data were used

PK data at 50 mg BID, 100 mg QD and 300 mg QD were used from the 15-day multiple-dosing cohort in a phase 1 study of ONO-5334 IR tablets (MAD study, *n* = 96) [31]. The MAD study was a randomized, double-blind, single-center study conducted at the Kendle Clinical Pharmacology Unit, Utrecht, The Netherlands, from April to November 2006 (EudraCT: not applicable). The sigmoidal relationship (*Emax* model) between plasma ONO-5334 concentrations and sCTX inhibition was taken from a study using SR tablets of ONO-5334 (PKPD study, *n* = 10) [29]. The PKPD study was a phase 1, 2-part (4- and 2-way crossover), open-label, randomized trial conducted at Pharmaceutical Profiles, Ruddington, Nottingham, United Kingdom, from January 2008 to April 2008 (European Union Clinical Trials Register [EudraCT]: 2007–005206-47). Data for sCTX inhibition and increase in BMD after 1 year of treatment were taken from the OCEAN study (*n* = 285) [16]. This study was a randomized, double-blind, multicenter study conducted at 13 sites in six European countries from December 2007 to August 2009 (EudraCT: 2007–002417-39, ClinicalTrials.gov Identifier: NCT00532337). The timing of 1 year was the end of the administration period in the original OCEAN study and the last point for PK data. Summaries of the two earlier phase I studies and the OCEAN study are given in Additional file 1.

In all studies, the protocol and consent form were reviewed and approved by an independent ethics committee prior to study initiation. Inclusion criteria for the MAD and PKPD studies were healthy postmenopausal females aged 45 to 75 years old with a body mass index of 19 to 32 kg/m^2^ [28, 31]. All participants had cessation of menstruation for more than 1 year before inclusion in the trials. The OCEAN study included postmenopausal women aged 55 to 75 years old with osteoporosis or osteopenia with one fragility fracture (at the start of the study), but otherwise in good general health [16]. Patients had cessation of menstruation for more than 5 years before inclusion in the trial. Osteoporosis was defined as a T-score ≤ −2.5 and osteopenia as a T-score ≤ −1 and > − 2.5 at the lumbar spine or total hip. Patients with urinary CTX <200 mg/mmol creatinine were excluded in the OCEAN study.

### Measurement of the plasma ONO-5334 concentration

Plasma ONO-5334 was determined by a liquid chromatography-tandem mass spectrometry with a lower limit of quantification of 0.02 ng/mL [18, 29]. The precision of the ONO-5334 assay was <15% (coefficient of variation) and the accuracy was within 15% of the actual value. In the 50 mg BID group in the MAD study, ONO-5334 was administered at 12-h dose intervals on days 1 to 14, and QD on day 15 (in the morning) to allow determination of terminal clearance similarly to other regimens. Complete PK data were available on days 1 and 15. A steady state plasma ONO-5334 concentration was reached on day 15; therefore, PK data from 0 to 12 h after dosing on day 15 were used and data from 12 to 24 h were substituted by values from 0 to 12 h.

### Measurement of the sCTX concentration

The level of sCTX was determined by a CrossLaps enzyme-linked immunosorbent assay (ELISA) at Charles River Laboratories (Edinburgh, UK) [16, 28]. Blood samples were collected in the morning after overnight fasting in the OCEAN study, but collected under a fed condition in the PKPD study. All CTX values used in simulations were higher than the lower limit of detection and the bottom of the standard curve for the assay.

### Simulation of sCTX levels from plasma ONO-5334 concentrations

**Fig. 1:**
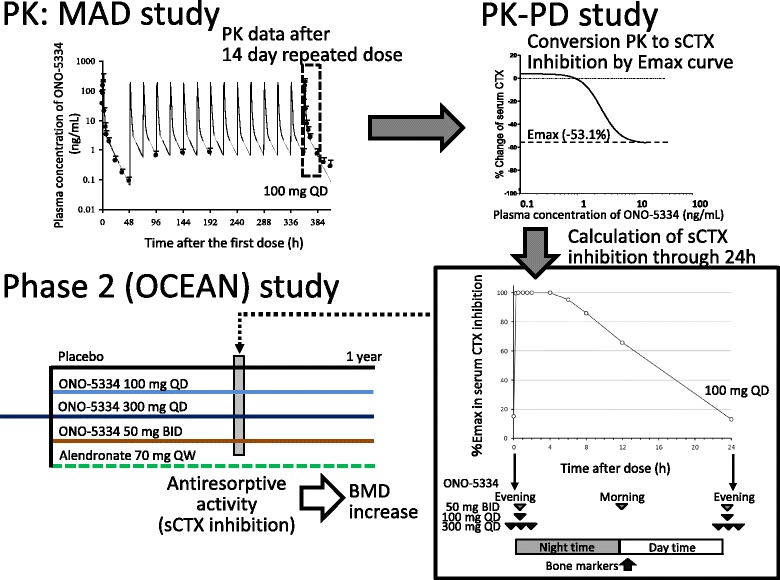
Simulation of serum CTX (sCTX) inhibition in the OCEAN study. Plasma ONO-5334 concentrations in postmenopausal women were measured in the MAD study [31]. Plasma ONO-5334 concentrations at doses of 50 mg BID, 100 mg QD and 300 mg QD were converted into simulated sCTX inhibition using a sigmoidal *Emax* model from a PK/PD study [29]. Simulated sCTX inhibition over 24 h for doses and regimens used in the OCEAN study (100 mg QD, 300 mg QD, and 50 mg BID) were then calculated [16]. Data for 100 mg QD is used as an illustrative example. BMD: bone mineral density

PK data from the MAD study and the relationship between plasma ONO-5334 levels and sCTX inhibition in healthy postmenopausal women obtained from the PKPD study were used in simulation of the antiresorptive effect of ONO-5334 [29, 31]. Our previous PK data for 14 days after repeated administration were also used in the simulation. sCTX inhibition for doses and regimens in the OCEAN study were simulated using plasma ONO-5334 levels and the sigmoidal *Emax* model in the PKPD study (Fig. 1). The sigmoidal *Emax* model was fitted to data using log-transformed ONO-5334 plasma concentrations and effects on sCTX inhibition, using the following equation [32].

$$ \boldsymbol{Effect}={\boldsymbol{E}}_0+\frac{{\boldsymbol{E}}_{\boldsymbol{\max}} \times {\boldsymbol{C}}^{\boldsymbol{r}}}{{\boldsymbol{E}\boldsymbol{C}}_{50}^{\boldsymbol{r}}+{\boldsymbol{C}}^{\boldsymbol{r}}} $$

where *C* is the plasma ONO-5334 concentration, the baseline effect (*E*_*0*_) is 0.9%, the effect span (*Emax*) is −54.0%, the half-maximal effective concentration of ONO-5334 (*EC*_*50*_) is 2.07 ng/mL, and the sigmoidicity factor (Hill constant, *γ*) is 2.07 for a negative change [29]. Mean sCTX inhibition over 24 h was calculated from the area under the sCTX inhibition (*%Emax*) curve (AUC) from 0 to 24 h after ONO-5334 administration, which was determined using the trapezoidal method. The mean sCTX inhibition (*%Emax*) over 24 h was calculated by dividing AUC by 24 h. The duration of sCTX inhibition over 24 h was evaluated for ≥90%, ≥70, ≥50 and ≥30% *Emax* using time intersections in the simulated sCTX inhibition (*%Emax*) plot. The 95% confidence interval (CI) was calculated for sCTX inhibition (*%Emax*) at 24 h after administration, but not for time-related analysis such as AUC and duration of sCTX inhibition.

### Relationship of sCTX inhibition and increase in BMD in the OCEAN study

The relationship of sCTX inhibition for 24 h and the mean increase in BMD for the lumbar spine (L1-L4) and total hip after 1 year of treatment in the OCEAN study were analyzed. BMD in the OCEAN study was measured using dual-energy X-ray absorptiometry (DXA) (Prodigy, GE Lunar, Madison, WI, USA) [16]. DXA images obtained at study sites were sent to a central site for image analysis (Synarc A/S, Zweigniederlassung, Hamburg, Germany). Linear regression analysis was used to evaluate correlations of mean sCTX inhibition (*%Emax*) over 24 h and BMD increases using Pearson correlation analysis (GraphPad Prism Software Inc., San Diego, CA), with significance defined as *p* < 0.05. The OCEAN study continued for 2 years of treatment with assessment of bone resorption markers and BMD [16, 17], but the last available PK data were 1 year after administration.

## Results

### Baseline characteristics

Age, weight, sCTX and other parameters at baseline in the MAD, PKPD and OCEAN studies are shown in Table 1. All studies were conducted in European countries and included only postmenopausal Caucasian women. Mean ages ranged from 60.6 to 65.3 years and mean baseline weights ranged from 65.4 to 71.1 kg, with slightly higher values in the MAD and PKPD studies than in the OCEAN study. Mean sCTX ranged from 0.550 to 0.898 ng/mL.

**Table 1:** Baseline Characteristics of the Study Population

Study	MAD^a^	PKPD^b^	OCEAN^d^
Subjects/Patients	Postmenopausal women	Postmenopausal women	Postmenopausal women with osteoporosis or osteopenia
Number of subjects/ patients	27	10	278
Race	100% Caucasian	100% Caucasian	100% Caucasian
Age (Mean (SD), year)	60.6 (5.7)	63.2 (4.7)	65.3 (4.8)
Weight (Mean (SD), kg)	69.8 (7.4)	71.1 (9.8)	65.4 (9.8)
Height (Mean (SD), cm)	165.2 (5.0)	160.3 (6.0)	160.1 (5.9)
BMI (Mean (SD), kg/m^2^)	25.6 (2.4)	27.6 (2.7)	25.5 (3.7)
Serum CTX (Mean (SD), ng/mL)	0.550 (0.209)	0.898 (0.171)^c^	0.739 (0.249)
Urine CTX (Mean (SD), μmol/mmol creatinine)	215 (91)	361 (165)	296 (128)

^a^Only 50 mg BID, 100 mg QD and 300 mg QD groups in 15-day cohort. The original study was reported by Nagase et al. [31]

^b^Value of all predose [28]

^c^Baseline data of the first period

^d^Full analysis set in the OCEAN study [16]

### Simulated sCTX inhibition based on plasma ONO-5334 concentration

**Fig. 2:**
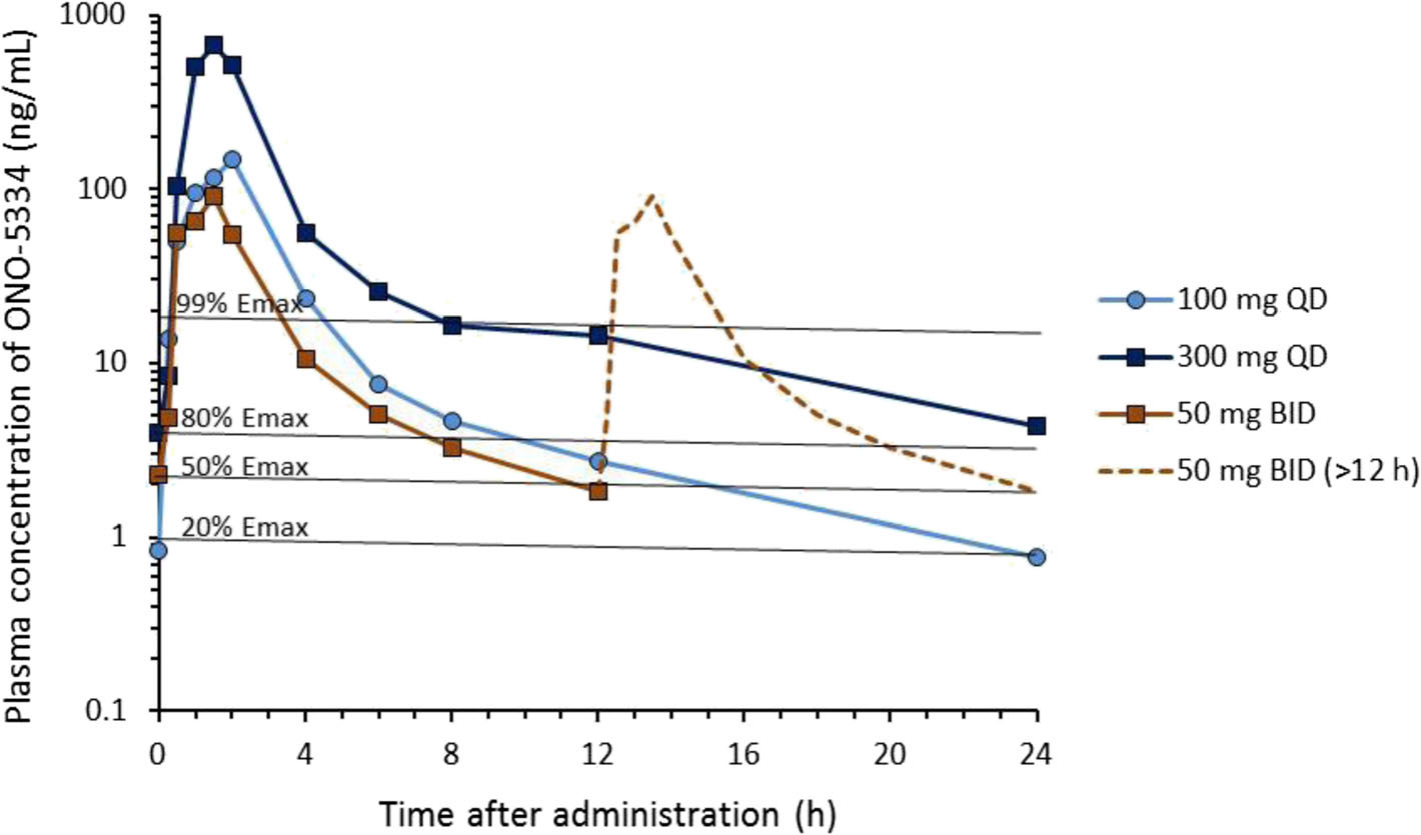
Plasma ONO-5334 concentrations following administration and those required for different levels of sCTX inhibition. Plasma ONO-5334 concentrations at 100 mg QD, 300 mg QD and 50 mg BID in the MAD study are shown. At 50 mg BID, plasma ONO-5334 concentrations between 12 and 24 h after administration are substituted by values between 0 and 12 h. ONO-5334 concentrations required for sCTX inhibition of 20, 50, 80 and 99% *Emax* are indicated. The plasma ONO-5334 concentration in the MAD study was reported by Nagase et al. [29]

**Fig. 3:**
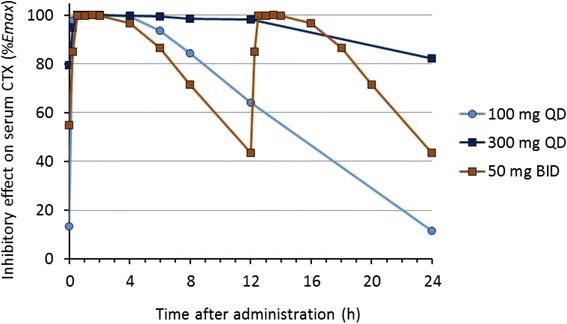
Simulated % *Emax* for sCTX inhibition over 24 h after ONO-5334 administration. Each symbol depicts sCTX inhibition obtained from the *Emax* equation using the plasma ONO-5334 concentrations shown in Fig. 2. *Emax* is the predicted maximum drug effect as 53.1% sCTX inhibition

**Fig. 4:**
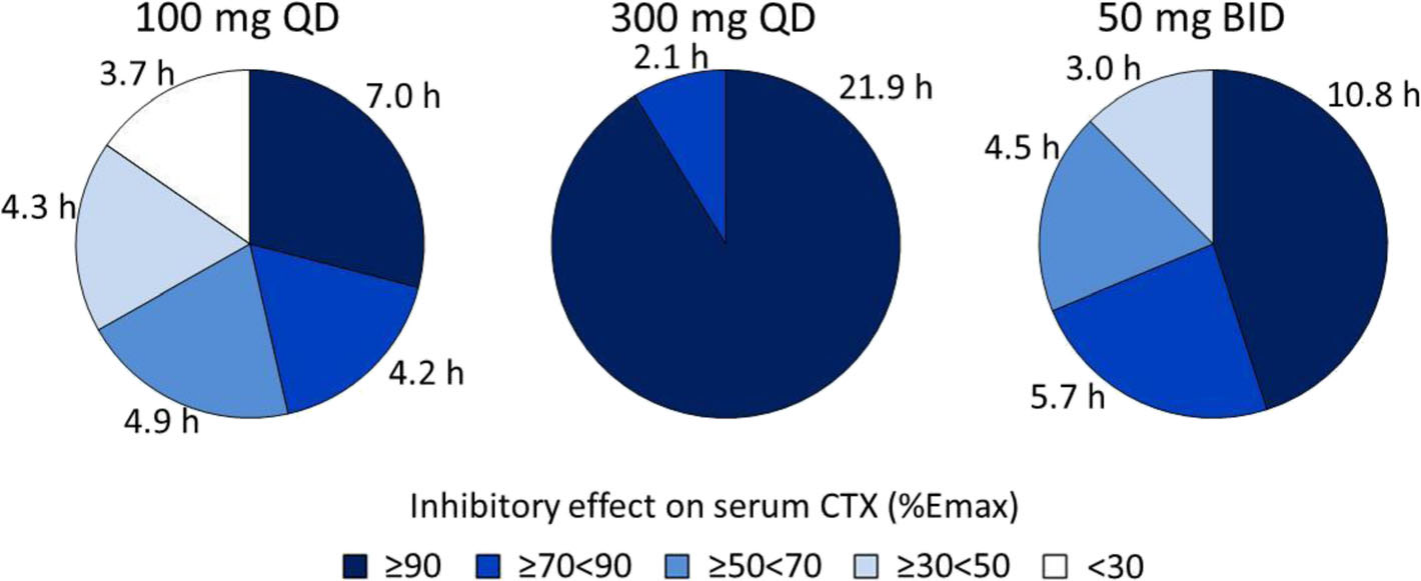
Duration of simulated % *Emax* for sCTX inhibition over 24 h evaluated for the following categories of *% Emax*: ≥90, ≥70, ≥50 and ≥30%

In accord with the rapid increase of the plasma ONO-5334 concentration, complete sCTX inhibition (≥99% *Emax*) was achieved 0.5 h after administration in all treatment groups (Figs. 2 and 3). From 1.5 to 2 h after administration, plasma ONO-5334 concentrations reached Cmax in the 100 mg QD, 300 mg QD and 50 mg BID groups and were 14, 56, and 4 times higher than the concentration required for 99% *Emax*, respectively. After Cmax, sCTX inhibition fell in accord with the rapid decrease in ONO-5334, and was <80% *Emax* at 100 mg QD and 50 mg BID at 12 and 8 h after administration, respectively. sCTX inhibition at 100 mg QD decreased to 12% *Emax* (95% CI: 0–50%) at 24 h after administration, but sCTX inhibition with 50 mg BID was 43% *Emax* (95% CI: 18–74%) at 12 h after the first dose and increased again at 12 h after the second dose in one day. sCTX inhibition at 300 mg QD was maintained at >98% *Emax* at 12 h after administration, and decreased to 82% *Emax* (95% CI: 24–100%) at 24 h. The duration of sCTX inhibition over 24 h was longer at 50 mg BID than at 100 mg QD for ≥90, ≥70, ≥50 and ≥30% *Emax* (Fig. 4), despite the total daily ONO-5334 dose being the same. The mean sCTX inhibition rates for 24 h at 100 mg QD, 300 mg QD and 50 mg BID were 63, 95 and 80% *Emax*, respectively.

### Comparison of simulated sCTX inhibition with observed data in the OCEAN study

Next, we compared simulated sCTX inhibition at 12 h after administration with observed sCTX inhibition in the OCEAN study [16]. The simulated sCTX inhibition rates at 100 mg QD, 300 mg QD and 50 mg BID were 35, 53 and 24% (66, 100 and 45% *Emax*), respectively. The observed sCTX inhibition rates (standard deviation) for 1 year treatment in the OCEAN study were 24% (40%), 44% (38%) and 29% (41%), respectively.

### Relationship between simulated sCTX inhibition and increase in BMD

**Fig. 5:**
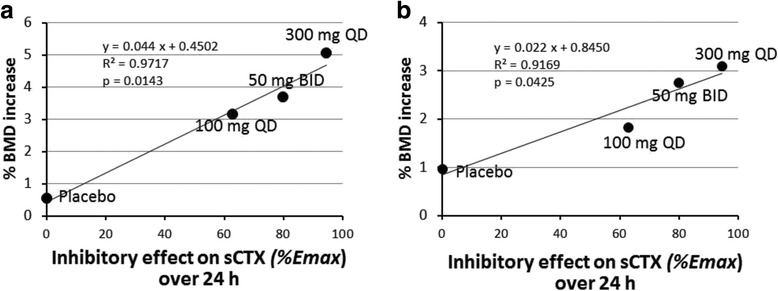
Relationships of mean sCTX inhibition over 24 h with observed increases in BMD over 1 year in the OCEAN study [16]. Relationships are shown for *% Emax* for sCTX inhibition with increases in (**a**) lumbar spine (LS1–4) BMD and (**b**) hip BMD. BMD data in the OCEAN study were reported by Eastell et al. [16]

The relationships between simulated sCTX inhibition over 24 h and observed increases in BMD of the lumbar spine and total hip in the OCEAN study at 1 year are shown in Fig. 5. BMD increases at both measured sites were positively correlated with sCTX inhibition over 24 h. The maximum BMD increases (100% *Emax* for 24 h) at 1 year were calculated by linear regression to be 4.85% in the lumbar spine and 3.05% in the total hip.

## Discussion

Simulated sCTX inhibition quickly reached >99% *Emax* at 0.5 h at all doses, but then fell below 80% *Emax* at 100 mg QD and 50 mg BID, but not at 300 mg QD. The mean sCTX inhibition rates over 24 h at 100 mg QD, 300 mg QD and 50 mg BID were 63, 95 and 80% *Emax*, respectively. The longest sCTX inhibition occurred with 300 mg QD, followed in order by 50 mg BID and 100 mg QD. The observed increases in BMD at the lumbar spine and total hip in the OCEAN study showed strong relationships with mean sCTX inhibition over 24 h. Taken together, these data show that the mean antiresorptive effects of ONO-5334 over one day at 100 mg QD and 50 mg BID were <90% *Emax*. However, 300 mg QD gives almost maximal potential of 96% *Emax* for antiresorptive effect. These results show that simulation of antiresorptive effect over 24 h allows prediction of BMD increases due to ONO-5334.

All three clinical studies used in this post-hoc analysis included only postmenopausal Caucasian women. The mean age of the patients in the OCEAN study was slightly higher than in the MAD study, and the mean body weight in the OCEAN study was 7% lower than in the MAD study. Plasma ONO-5334 levels in the OCEAN study were comparable to those in the MAD study (Additional file 2). There were differences in baseline levels of bone resorption markers among studies, but these levels do not seem to influence the antiresorptive effect of cathepsin K inhibitors [16, 25]. Consequently, the slight differences in baseline characteristics were considered not to be clinically relevant or to have significantly affected the outcome of the current analysis. Therefore, it was considered appropriate to estimate the antiresorptive effect of ONO-5334 in the OCEAN study based on data from the MAD and PKPD studies.

The timing of administration also differed among studies. ONO-5334 was administered in the morning in the MAD and PKPD studies, but the QD groups received ONO-5334 in the evening in the OCEAN study. PK is influenced by variation of physiological functions with time of day [33, 34]. Among these factors, gastric pH may influence absorption of ONO-5334 because the solubility of ONO-5334 is high at acidic pH. Gastric pH transiently increases from pH 2 to pH 4 after a meal [35], although PK parameters, Cmax and AUC of ONO-5334 do not differ significantly in postprandial administration compared with a fasted state [18]. Dissolution of IR tablets is rapid (50% dissolution in vitro in <0.25 h). Therefore, the difference in timing of administration with ONO-5334 was unlikely to have significantly influenced the PK. However, the timing of administration may influence antiresorptive effects due to circadian rhythms in bone turnover, which reach a peak during the night/early morning and a nadir in the late afternoon [26, 27]. Eastell et al. showed that changes in sCTX inhibition with ONO-5334 SR in morning vs. evening dosing parallel changes in PK [30]. In addition, the sigmoidal *Emax* model of sCTX with ONO-5334 SR was similar under fed and fasted conditions [29]. Overall, these limitation in differences in PK are unlikely to have significantly influenced the simulation of levels of bone resorption markers.

This post-hoc analysis showed that the mean antiresorptive effect over 24 h had a significant positive relationship with observed increases in BMD in the OCEAN study. It is difficult to explain why the increase in BMD at 50 mg BID was higher than that at 100 mg QD from the observed sCTX and PK data in the OCEAN study, in which there was no marked difference in sCTX between the two groups at 12 months. However, the simulation of antiresorptive effect at 50 mg BID clearly showed higher sCTX inhibition compared with 100 mg QD, and thus may help to explain the change in BMD relative to changes in bone resorption. ONO-5334 SR can reduce excessive exposure and improve adherence by reducing dosing frequency from BID to QD. On the other hand, 300 mg QD was the most effective dose and regimen for a BMD increase in the OCEAN study, and had no safety concerns, even though Cmax was 74 times higher than that required for 99% *Emax* [16, 17]. In the current analysis, the antiresorptive effect over 24 h at 300 mg QD almost reached maximum inhibition. Several cathepsin K inhibitors have been reported [18, 19, 36, 37] and these have different PK and safety profiles [18, 25]. However, the maximal effects of drugs in the same class may not differ [38] and the effect of 300 mg QD ONO-5334 may reflect the maximal effect of cathepsin K inhibitors on BMD. The maximal BMD increase with ONO-5334 was not less than that observed for odanacatib at 50 mg once weekly [16, 23].

This study has a number of strengths, including use of a PK/PD analysis to evaluate specific regimens, dose response, and maximal response in the OCEAN study. However, there are also several limitations. First, this was a post-hoc analysis that used data that were not designed for the analysis. Second, there is a time mismatch between data at 2 weeks after repeated administration in the simulation and observed 1-year data in the OCEAN study. However, this mismatch was considered minimal because the antiresorptive effect and PK of ONO-5334 were stable and BMD gradually increased over 1 year [16]. Third, the inhibitory effect on osteoblast function, e.g. P1NP, of cathepsin K inhibitors is slightly less than that of bisphosphonates [16]. This is an important feature of cathepsin K inhibitors, but we used only antiresorptive effect to simplify the PK/PD analysis.

## Conclusion

This study shows that simulation of antiresorptive effect via sCTX inhibition provides a quantitative prediction of the dose- and regimen-dependent effects of ONO-5334. In addition, the analysis clarified the relationship of antiresorptive effect over 24 h with BMD increase. The simulation confirmed the observation that a dose of 50 mg BID is more effective than 100 mg QD (the same total daily dose) for increasing BMD and suppressing antiresorptive effect. Thus, simulation of antiresorptive effect over 24 h permits prediction of the BMD increase induced by ONO-5334. This is important because sustained release preparations may have better compliance and maximize antiresorptive potency using a lower dose of ONO-5334.

## Additional files

Additional file 1: Table S1. Study design, dosing duration, dosage strength, regimen and formulation of OCEAN, PKPD and PK studies. (DOCX 71 kb)

Additional file 2: Table S2. Plasma ONO-5334 concentrations in MAD and OCEAN studies. (DOCX 70 kb)

Additional file 3: Table S3. Independent ethics committees of OCEAN, PKPD and PK studies. (DOCX 45 kb)

## Abbreviations

AUC: Area under the curve
BID: Twice daily
BMD: Bone mineral density
Cmax: Higher maximum level
CTX: C-telopeptides of type I collagen
DXA: Dual-energy X-ray absorptiometry
*Emax*: Sigmoidal maximal effect
IR: Immediate release
NTX: N-telopeptides of type I collagen
PK: Pharmacokinetic
QD: Once daily
sCTX: Serum CTX
SR: Slow release
