# Supplementary material for: Antiresorptive effect of a cathepsin K inhibitor ONO-5334 and its relationship to BMD increase in a phase II trial for postmenopausal osteoporosis
Source: BMC Musculoskelet Disord. 2017 Jun 19;18:267. doi: 10.1186/s12891-017-1625-y (PMC5477094; doi:10.1186/s12891-017-1625-y)
Supplement: Supplementary file 1 — Study design, dosing duration, dosage strength, regimen and formulation of OCEAN, PKPD and PK studies. (DOCX 71 kb) [file 12891_2017_1625_MOESM1_ESM.docx]

**Additional file 1** Studies of Data Used

| Study | MAD^a^ | PKPD^b^ | OCEAN (1 year)^c^ |
| --- | --- | --- | --- |
| Use in the study | Source of PK data | Conversion PK to PD | Simulated and reference |
| Phase | Phase I | Phase I | Phase II |
| Design | randomized, double blinded, single center | randomized, open label,  4 + 2 way crossover,  single center | randomized, double blinded,  multi center |
| Dosing duration | 15-day multiple dose after single dose | Single dose | 1 year multiple dose (Extension :  +1 year multiple dose) |
| Dosage strength, regimen (timing) | 50 mg BID (morning & evening)  100 mg QD (morning)  300 mg QD (morning) | 100 mg (morning) | 50 mg BID (morning & evening)  100 mg QD (evening)  300 mg QD (evening) |
| Formulation used | IR tablets | SR tablets (SR granules^#^) | IR tablets |

a: Only groups of 100 mg QD, 300 mg QD, 50 mg BID in 15-day cohort were used from data reported by Nagase et al [31].

b: Data was reported by Tanaka et al [28].

c: Data of the original OCEAN study of 1 year was used, instead the study was extend additional 1year reported by Eastell et al [16].

PK: pharmacokinetic, PD: pharmacodynamic, IR: immediate release, SR: sustained release

#: SR granules were not used in PKPD analysis because lower level in Cmax
