# Supplementary material for: Antiresorptive effect of a cathepsin K inhibitor ONO-5334 and its relationship to BMD increase in a phase II trial for postmenopausal osteoporosis
Source: BMC Musculoskelet Disord. 2017 Jun 19;18:267. doi: 10.1186/s12891-017-1625-y (PMC5477094; doi:10.1186/s12891-017-1625-y)
Supplement: Supplementary file 2 — Plasma ONO-5334 concentrations in MAD and OCEAN studies. (DOCX 70 kb) [file 12891_2017_1625_MOESM2_ESM.docx]

**Addional file 2** Plasma ONO-5334 concentrations

|  | 50 mg BID | 100 mg QD | 300 mg QD |
| --- | --- | --- | --- |
| **Plasma ONO-5334 concentrations (ng/mL)** | | | |
| At 12 h after administration in MAD study (Mean ± SD)^a^ | 1.82 ± 0.33 | 2.74 ± 0.99 | 14.5 ± 6.33 |
| At morning on 12 month in the OCEAN study (Mean ± SD)^b^ | 2.51 ± 1.60 | 4.51 ± 3.26 | 14.4 ± 10.6 |

a: data from the MAD study [31].

b: full analysis set in the OCEAN study [16].
