# Supplementary material for: Antiresorptive effect of a cathepsin K inhibitor ONO-5334 and its relationship to BMD increase in a phase II trial for postmenopausal osteoporosis
Source: BMC Musculoskelet Disord. 2017 Jun 19;18:267. doi: 10.1186/s12891-017-1625-y (PMC5477094; doi:10.1186/s12891-017-1625-y)
Supplement: Supplementary file 3 — Independent ethics committees of OCEAN, PKPD and PK studies. (DOCX 45 kb) [file 12891_2017_1625_MOESM3_ESM.docx]

**Additional file 3:** Independent ethics committees

OCEAN study

| De Videnskabsetiske Komiteer for Region Hovedstade, Hillerod, Denmark |
| --- |
| EgeszsegOgyi Tudomanyos Tanacs, Budapest, Hungary |
| Ethics Commitee for Mu!tlcentric Clinical Trials, Prague, Czech Replublic |
| Independent Review Board, Amsterdam, The Netherlands |
| Lithunian Bioethics Committee, Vilnius, Lithuania |
| Tallinn Medical Research Ethics Committee, Tallinn, Estonia |

PKPD study

| Capenhurst Independent Research Ethics Committee, Wirral, United Kingdom |
| --- |

MAD study

| Stichting Therapeutische Evaluatie Geneesmiddelen, Almere, The Netherlands |
| --- |
